# Optogenetic ion pumps differ with respect to the secondary pattern of K^+^ redistribution

**DOI:** 10.14814/phy2.15778

**Published:** 2023-08-03

**Authors:** R. Ryley Parrish, Tom Jackson‐Taylor, Juha Voipio, Andrew J. Trevelyan

**Affiliations:** ^1^ Medical School, Newcastle University Biosciences Institute Newcastle upon Tyne UK; ^2^ Department of Cell Biology and Physiology Brigham Young University Provo Utah USA; ^3^ Faculty of Biological and Environmental Sciences, Molecular and Integrative Biosciences University of Helsinki Helsinki Finland

**Keywords:** archaerhodopsin, chloride, chloride‐cation‐cotransporter, halorhodopsin, potassium

## Abstract

We recently reported that strong activation of the optogenetic chloride pump, halorhodopsin leads to a secondary redistribution of K^+^ ions into the cell, through tonically open, “leak” K^+^ channels. Here we show that this effect is not unique to halorhodopsin but is also seen with activation of another electrogenic ion pump, archaerhodopsin. The two opsins differ however in the size of the rebound rise in extracellular potassium, [K^+^]_o_, after the end of activation, which is far larger with halorhodopsin than for archaerhodopsin activation. Multiple linear regression modeling indicates that the variance in the postillumination surge in [K^+^]_o_ was explained both by the size of the preceding, illumination‐induced drop in [K^+^]_o_ and also by the type of opsin. These data provide additional support for the hypothesis that intense chloride‐loading of cells, as occurs naturally following intense bursts of GABAergic synaptic bombardment, or artificially following halorhodopsin activation, is followed by extrusion of both Cl^−^ and K^+^ coupled together. We discuss this with respect to the pattern of [K^+^]_o_ rise that occurs at the onset of seizure‐like events.

## 1 INTRODUCTION

The development of optogenetics techniques and tools has presented new ways of manipulating neuronal excitability (Deisseroth, 2015), using either light‐activated ion channels (Boyden et al., 2005) or electrogenic ion pumps (Chow et al., 2010; Zhang et al., 2007). The real power of these remarkable tools is that they may be targeted very precisely to specific subsets of cells, or even to subcellular domains, allowing experimental control to be delivered at these sites. The induced ion movements, however, do alter the internal and external milieu, meaning that there may be unintended secondary effects both at and away from the site of expression. These, though, may be put to good use for exploring neuronal physiology and pathology, so it is worth documenting these effects.

Halorhodopsin was the first optogenetic protein to be used for inhibiting neurons, using light to drive chloride into neurons to hyperpolarize them (Zhang et al., 2007). We recently showed that this light‐activated chloride movement has several interesting secondary effects (Parrish et al., 2023). Firstly, the hyperpolarization of membrane potential alters the balance between the electrical and chemical gradients for other ions; since the open “leak” channels at rest are mainly K^+^ channels, there follows a redistribution of K^+^ ions into expressing cells, causing the extracellular K^+^ concentration, [K^+^]_o_, to drop. Consequently, there is a negative shift in membrane potential in other non‐expressing neurons, with a measurable drop in their excitability (an “off‐target” inhibition that may be important to recognize when interpreting optogenetic experiments). Most surprising, though, was that activation of halorhodopsin triggered cortical spreading depolarization (CSD) events that occurred even during periods of ongoing illumination, when most neurons were hyperpolarized and [K^+^]_o_ was below baseline levels. Interestingly, in the trials where CSD was not induced and once illumination ended, there was then a large rebound surge in [K^+^]_o_.

Archaerhodopsin, another commonly used inhibitory optogenetic ion pump, delivers hyperpolarization of neurons in a different way, by driving protons out of the cell (Chow et al., 2010). As such, archaerhodopsin activation should show some of the secondary effects of halorhodopsin, but not others. It has already been shown that activation of halorhodopsin, but not archaerhodopin, induces a pronounced chloride‐loading of neurons that may persist for many seconds (Alfonsa et al., 2015; Raimondo et al., 2012). Since Cl^−^ clearance is achieved primarily by coupling to K^+^ extrusion, through the electroneutral chloride‐cation cotransporter, KCC2 (Kaila et al., 2014; Rivera et al., 1999), the degree of chloride loading will influence the amplitude of the post‐illumination surge in [K^+^]_o_. To examine these differences, we made recordings of [K^+^]_o_ in neocortical brain slices in which archaerhodopsin is expressed widely in the pyramidal population, to show that it shares with halorhodopsin the effect of inducing an inward movement of K^+^ ions during the period of illumination. In contrast, the post‐illumination rebound surge in [K^+^]_o_ following archaerhodopsin activation appears much smaller than for halorhodopsin.

## 2 METHODS

### 2.1 Archaerhodopsin viral injections

C57/B6 adult mice were injected with AAV9.CAG.ArchT, purchased from the UPenn vector core. For these adult injections, done at 8–12 weeks of age, animals were anesthetized by ketamine–methoxamine intraperitoneal injection and placed in a stereotaxic head holder (David Kopf Instruments). Injections were made at three to four locations in an anterior–posterior row in one hemisphere, 1.5–2 mm lateral to the midline and 1–0.4 mm deep to the pia (0.6 μL, injected over 15 min).

### 2.2 Slice preparation

We used both wild‐type C57/B6 mice, and also mice expressing eNpHR3.0 within the pyramidal cell population, generated by cross‐breeding homozygous Emx1‐cre mice (Jackson Laboratory; Stock 005628) with mice containing a floxed STOP cassette in front of an eNpHR3.0/EYFP domain (Jackson Laboratory; Stock 014539; both maintained on the C57/B6 background). Emx1‐promoter yields gene expression only in pyramidal cells in neocortex, in adult mice, although it can drive genes in glia in other brain areas, and early in development (Gorski et al., 2002). Experiments were performed on mice aged 1–8 months, of both sexes. Mice were first decapitated, brains were removed, and placed in a cold cutting solution containing (in mM): 3 MgCl_2_; 126 NaCl; 2.6 NaHCO_3_; 3.5 KCl; 1.26 NaH_2_PO_4_; 10 glucose. 400 μm horizontal sections were made on a Leica VT1200 vibratome (Leica Microsystem). Slices were stored at room temperature, in an interface holding chamber for 1–4 h prior to experimentation. Solutions were bubbled with carboxygen (95% O_2_ and 5% CO_2_) in artificial cerebrospinal fluid (aCSF) containing (in mM): 2 CaCl_2_; 1 MgCl_2_; 126 NaCl; 26 NaHCO_3_; 3.5 KCl; 1.26 NaH_2_PO_4_; 10 glucose.

### 2.3 In vitro extracellular recordings

Extracellular recordings were performed using an interface recording chamber. Slices were placed in the recording chamber perfused with either aCSF, supplemented in different experiments with different combinations of the following drugs: 1 μM tetrodotoxin (TTX; Abcam), 10 mM tetraethylammonium (TEA; Sigma), 10 μM VU 0463271 (Tocris Bioscience), as indicated in the results section. Recordings were obtained using aCSF‐filled ~1–3 MΩ borosilicate glass microelectrodes (GC120TF‐10; Harvard Apparatus) placed in deep layers of neocortex. Extracellular potassium [K^+^]_o_ was measured using single‐barrelled K^+^‐selective microelectrodes. The pipettes were pulled from nonfilamented borosilicate glass (Harvard Apparatus), and the glass was exposed to the vapor of dimethyl‐trimethyl‐silylamine (Sigma‐Aldrich), baking at 200°C for 40 min. The pipettes were then backfilled with aCSF. A short column of the K^+^ sensor (Potassium ionophore I, cocktail B; Sigma‐Aldrich, #99373) was taken into the tip of the salinized pipette by using slight suction. The recordings through the K^+^‐sensor electrode were referenced to a second electrode filled with aCSF. From the differential signal from a custom build amplifier, we calculated the [K^+^]_o_ from calibration recordings made in an open bath, using sudden increments in [K^+^]_o_. This provided a scaling factor S, of 55–59 mV, where the K^+^ concentration at a given moment in time, *t*, was calculated from the differential voltage, *V*(*t*), as follows: ${\left[K\right]}_{o}={\left[K\right]}_{o.\text{baseline}}\;10^{V\left(t\right)/S}\;{\left[K\right]}_{o.\text{baseline}}$ for our experiments was 3.5 mM. The temperature of the chamber and perfusate was maintained at 33–36°C using a closed circulating heater (FH16D; Grant Instruments). The solutions were perfused at the rate of 3 mL/min by a Watson Marlow 501U peristaltic pump (Watson‐Marlow Pumps Limited). For the collection of the halorhodopsin and archaerhodopsin data, the direct current local field potential signal was unfiltered and amplified to a 10× output with a custom build amplifier. These waveform signals were digitized with a Micro 1401‐3 ADC board (Cambridge Electronic Design) and Spike2 version 7.10 software (Cambridge Electronic Design). Signals were sampled at 10 kHz.

Recordings were analyzed using a custom‐written code in Matlab2015b (Mathworks).

### 2.4 Optogenetic illumination for extracellular recordings

Optogenetic illumination was delivered for 90 s continuous periods, using a Fiber‐coupled LED light at 565 nm (Thorlabs; M565F3) and driven by an LED driver (Thorlabs; LEDD1) placed in trigger mode. Light output was measured at an average of 9 mW. The LED light was positioned just above the superficial layers of the neocortex for the slice experiments and pointed toward the deep layers, where the extracellular recordings were performed.

### 2.5 Statistics

Statistical analysis of electrophysiology was performed using GraphPad Prism (GraphPad Software, Inc.). Data were analyzed using MATLAB software (Mathworks) to implement Mann–Whitney test (*ranksum.m*), multiple linear regression modeling (*fitlm.m*), and analysis of covariance (*aoctool.m*) Data and the software code are available upon request.

## 3 RESULTS

### 3.1 Activation of archaerhodopsin causes secondary redistribution of K^+^ ions

**FIGURE 1:**
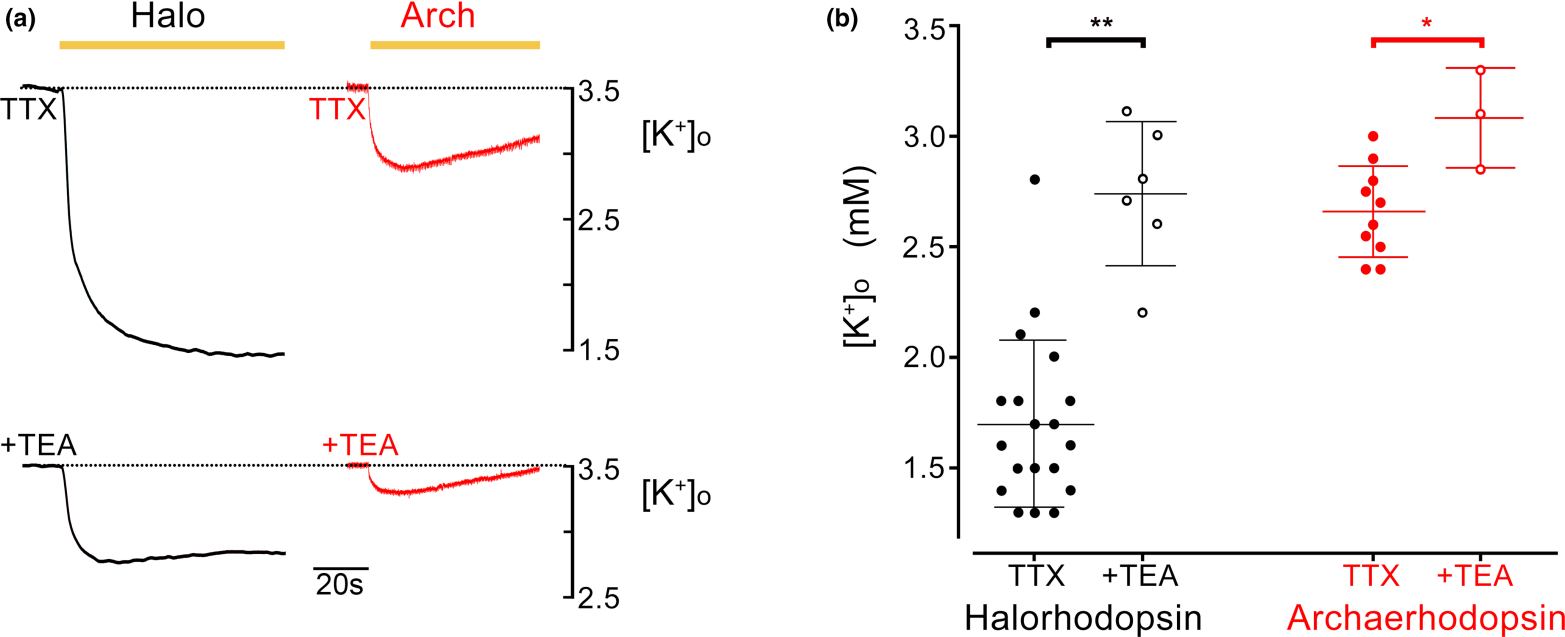
The main movement of K^+^ ions from the extracellular space, induced by opsin activation is through K^+^ channels. (a) Redistribution of K^+^, assessed by recording [K^+^]_o_ during a period of halorhodopsin (black) or of archaerhodopsin activation (red) in the presence of TTX to block neuronal firing, and additionally TEA to block “leak” K^+^ channels. (b) Pooled data of measurements of [K^+^]_o_ for all the halorhodopsin and archaerhodopsin recordings. For both opsins, the size of the K^+^ redistribution is greatly reduced by block of voltage‐gated K^+^ channels (Halo vs. Halo + TEA, **p = 0.00076; Arch vs. Arch + TEA, *p = 0.0279, two‐tailed Mann–Whitney test). TEA, tetraethylammonium; TTX, tetrodotoxin.

We previously reported that activation of the optogenetic chloride pump, halorhodopsin, induced a secondary ionic redistribution of K^+^ ions into the cell during the period of activation, followed by a surge in [K^+^]_o_ after illumination ended (Parrish et al., 2023). The inward movement of K^+^ appears to pass through leak K^+^ channels, whereas the post‐illumination outward movement included a KCC2‐sensitive component, implying that it was driven in part by the need to remove Cl^−^ ions that had been pumped into cells. We reasoned therefore that these effects may be replicated in part by activation of other optogenetic pumps. To test this prediction, we recorded the pattern of K^+^ redistribution associated with the activation of the proton pump, archaerhodopsin (Figure 1). Widespread archaerhodopsin expression in the pyramidal cell population was achieved by injecting a viral vector carrying the ArchT gene under the CAG promoter into wild‐type mice. During periods of illumination, we recorded a highly significant drop in [K^+^]_o_ in brain slices expressing archaerhodopsin (2.70 ± 0.08 mM [mean ± SEM], range 2.2–3.0 mM; 95% confidence interval = 2.55–2.85 mM; baseline [K^+^]_o_ = 3.5 mM; Figure 1), but not in brain slices from wild‐type mice. The archaerhodopsin‐induced drop was smaller than we had previously shown for halorhodopsin‐expressing brain slices (1.71 ± 0.08 mM, range 1.3–2.8 mM; 95% confidence interval = 1.55–1.88 mM; data reproduced here from Parrish et al., 2023), although this difference is likely explained by the weaker expression of archaerhodopsin, driven by viral vectors, compared with halorhodopsin, which was achieved by breeding transgenic lines. This archaerhodopsin‐driven K^+^ redistribution was reduced by 61% by blockade of plasmalemmal K^+^ channels, using TEA (*n* = 3; without TEA = 2.53 mM; with TEA = 3.12 mM), comparable to the 66% reduction of the halorhodopsin effect. In both respects, archaerhodopsin mimicked the effect we had previously reported in halorhodopsin‐expressing brain slices. In our preparations, the amplitude of the K^+^ redistribution was larger in the halorhodopsin‐expressing brain slices, which might reflect the degree of hyperpolarization achieved.

**FIGURE 2:**
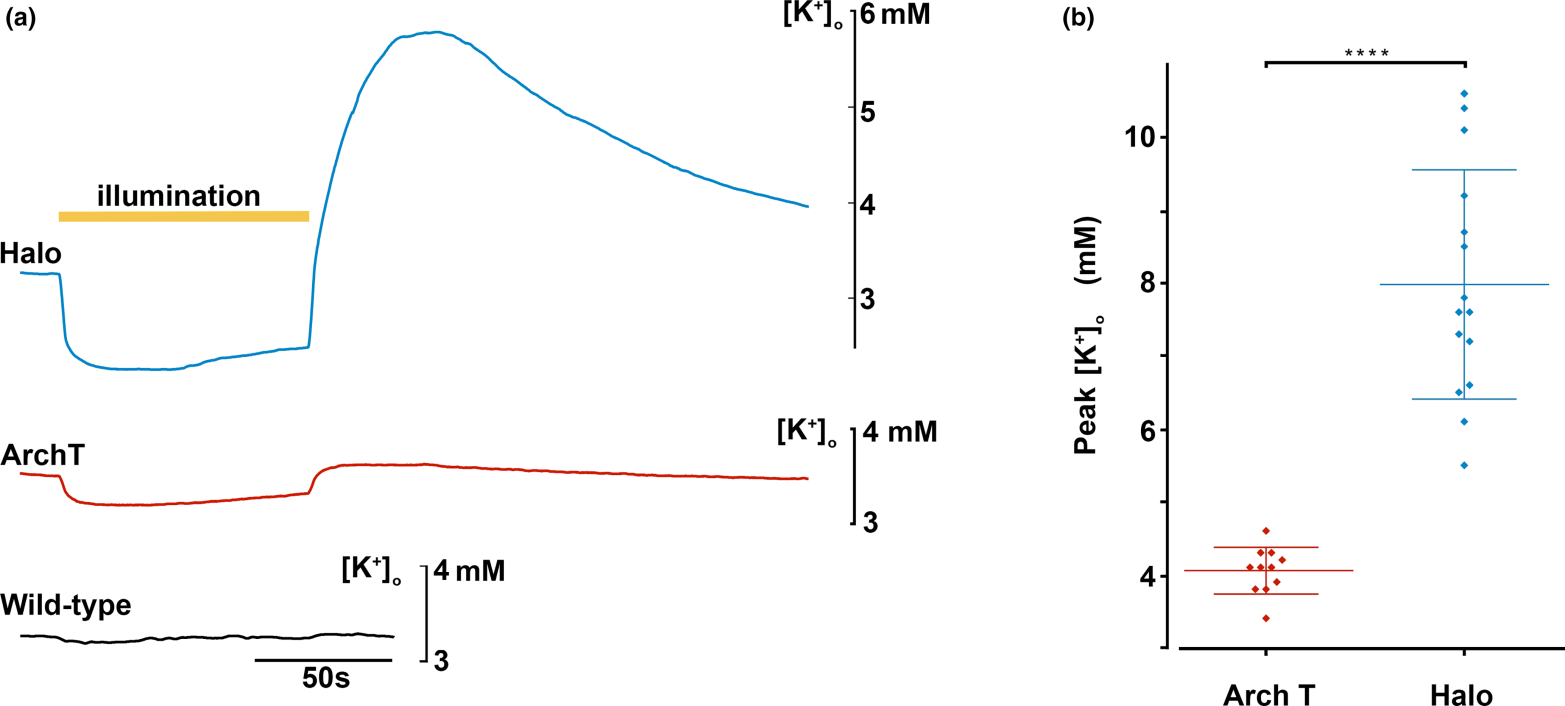
The large post‐illumination surge in [K^+^]_o_ following a period of halorhodopsin activation is not replicated by archaerhodopsin activation. (a) Recordings of extracellular [K^+^] in brain slices in which either halorhodopsin (blue, top), or archaerhodopsin (red, middle), was expressed in all pyramidal cells, or in a slice with no opsin expression (black, bottom). The yellow bar represents the time of illumination (90 s period). Light activation of halorhodopsin induced a very marked reduction in [K^+^]_o_, followed by an even larger, rebound increase. There was also a drop in [K^+^]_o_, synchronous with the activation of archaerhodopsin, but the rebound was negligible. Brain slices without opsins showed no light induced fluctuations in [K^+^]_o_. (b) The peak extracellular [K^+^], after the end of illumination, for archaerhodopsin (mean peak = 4.12 mM, *n* = 11) and halorhodopsin activation (mean peak = 7.98 mM; *n* = 15; comparison, ****p = 0.0001, two‐tailed Mann–Whitey test), respectively.

At the end of the illumination period, we previously reported that halorhodopsin‐expressing brain slices showed a very large rebound surge in [K^+^]_o_ (7.98 ± 0.41 [mean ± SEM], range 5.5–10.6 mM; 95% confidence interval = 7.18–8.78 mM). In contrast, the post‐illumination surge in archaerhodopsin‐expressing slices was significantly above the 3.5 mM baseline level (4.12 ± 0.13 mM, range 3.4–5.1 mM; 95% confidence interval = 3.86–4.38 mM), but significantly smaller than occurred with the halorhodopsin slices (archaerhodopsin vs. halorhodopsin, *p* = 0.0001, two‐tailed Mann–Whitey test) (Figure 2).

**FIGURE 3:**
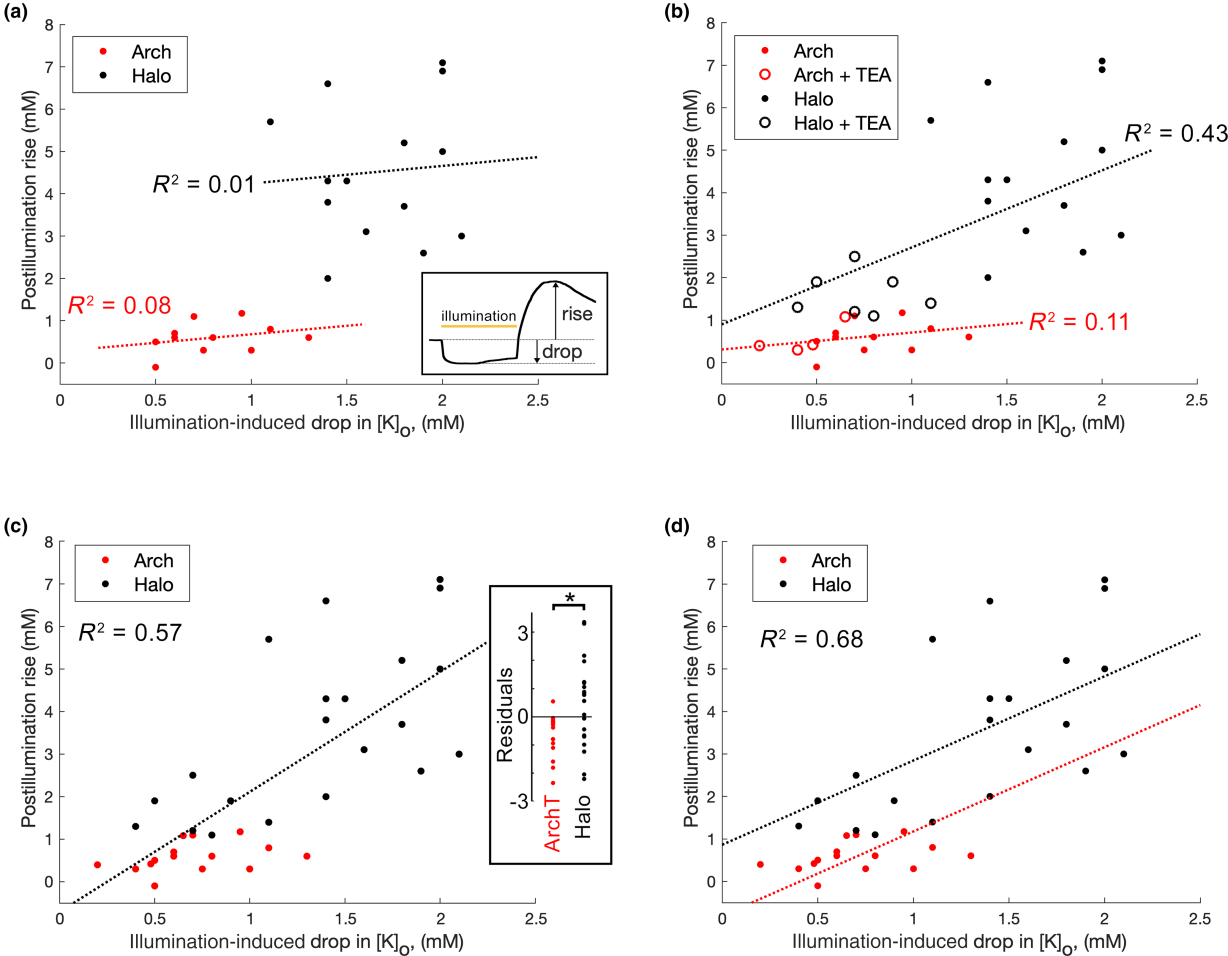
Linear regression modeling indicates that the post‐illumination [K^+^]_o_ rise depends upon both the drop in [K^+^]_o_ during illumination and also the opsin type. (a) The plot of the [K^+^]_o_ rise, in excess of baseline (3.5 mM), following the end of illumination versus the drop in [K^+^]_o_ below baseline that occurs during illumination (illustrated in inset). Data are shown for 25 different brain slices, 14 expressing halorhodopsin (red) and 11 expressing archaerhodopsin‐T (black) in the absence of modulating drugs. (b) The same plot, including data from TEA treated slices, which reduces the illumination driven influx of K^+^, and thereby extends the range of independent variables (the drop in [K^+^]_o_ during illumination). In both (a, b), the regressions and *R* ^2^ values for the two opsins are independent. (c) The same data as (b), without distinguishing between opsin types. The inset shows the residuals, for this best fit across all data; there is a highly significant difference between the opsins (*p = 0.0082, Mann–Whitney rank sum test). (d) The same data, showing the optimal model, in which the opsin type is treated as independent categorical groups (the relationship to the [K^+^]_o_ drop, for both opsins, is considered independent, and so the slopes are identical).

For both opsins, a component of the rebound surge will be extrusion of K^+^ ions that moved into the neurons during the period of illumination; since the illumination‐induced inward movement was greater in our halorhodopsin group (the range of the drop in of drop in [K^+^]_o_ = 1.2–2.2 mM below baseline (3.5 mM); during archaerhodopsin activation, the range = 0.5–1.3 mM below baseline), one possibility is that the larger rebound in that group simply reflected this difference. One approach to test this would have been to adjust expression patterns or illumination levels to try to match the amplitude of the illumination‐induced drop for the two different opsins, but the halorhodopsin experiments were performed first, and we were not able to achieve the same degree of K^+^ redistribution with the archaerhodopsin. Instead, we pooled the datasets and used multilinear regression modeling of the [K^+^]_o_, with the predictive variables being the illumination‐induced drop, and the type of opsin expressed, to assess their relative contributions to the post‐illumination surge (the dependent variable; Figure 3; Table 1).

**TABLE 1:** Linear regression model analyses of archaerhodopsin and halorhodopsin [K^+^]_extra_.

		Figure panel	*R* ^2^	*p* Value
[K^+^] rise vs.	[K^+^] drop (ArchT only)	3b (red)	0.111	0.207
	[K^+^] drop (Halo only)	3b (black)	0.426	0.010
	[K^+^] drop (both opsins pooled)	3c	0.566	<10^−7^
	[K^+^] drop, with opsins grouped	3d	0.678	<10^−8^

*Note*: Linear regression model analyses of the data presented in Figure 3, examining different models of the post‐illumination rise in [K^+^]_extra_ predicted by the expression of either halorhodopsin or ArchT and the degree of [K^+^]_extra_ drop during the period of illumination.

**FIGURE 4:**
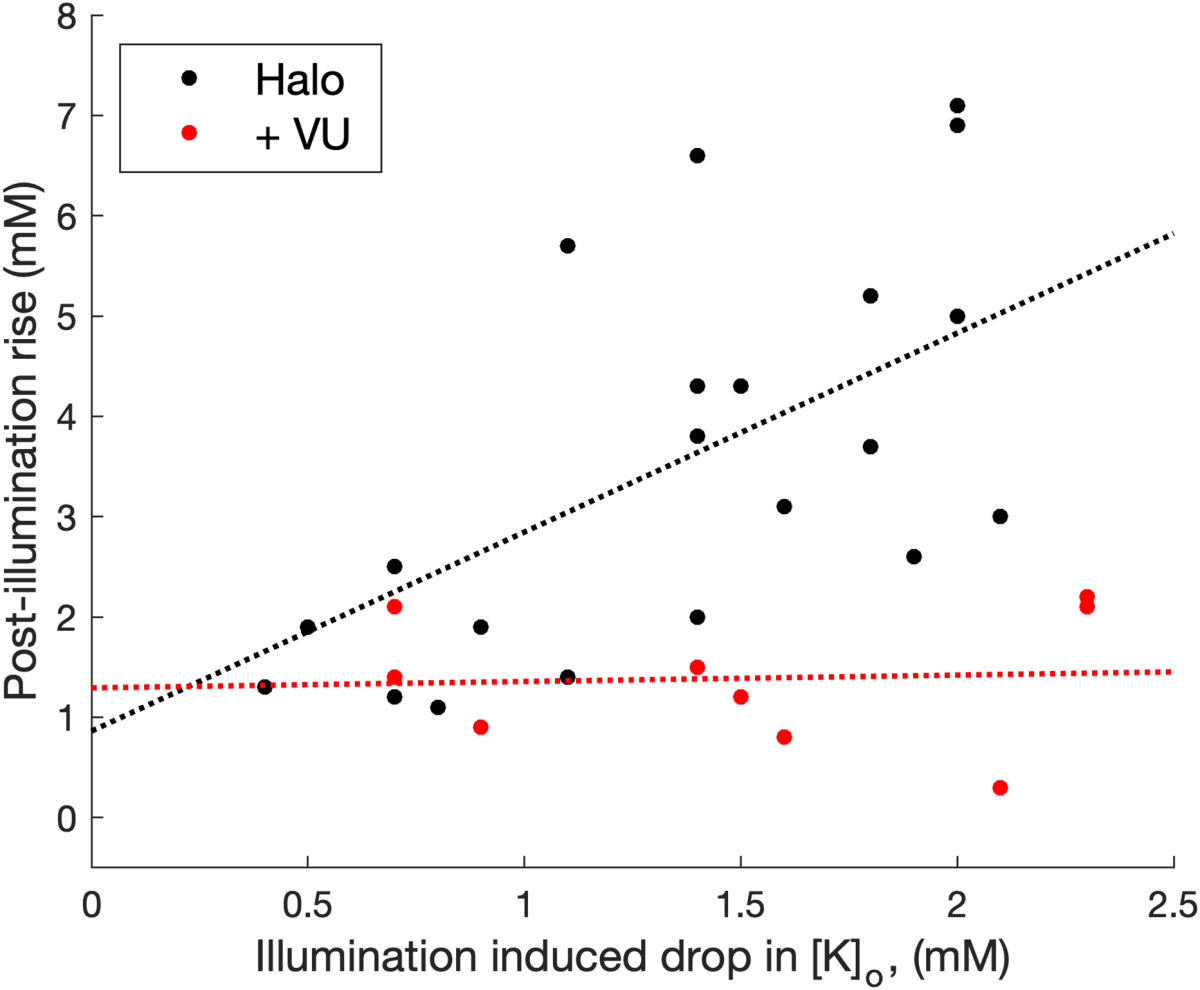
Decoupling of post‐illumination surge in [K^+^]_o_ by application of the KCC2 blocker, VU 0463271. Plots of the illumination‐induced [K^+^]_o_ drop, and the post‐illumination [K^+^]_o_ rise, in brain slices expressing halorhodopsin. Following the application of VU 0463271, the post‐illumination surge in [K^+^]_o_ is small and uncorrelated with the [K^+^]_o_ drop during the period of illumination (*R* ^2^ = 0.004; *p* = 0.87).

Our initial exploration was of the data set without pharmacological manipulations (Figure 3a), which showed very weak correlations between the illumination‐induced drop and the post‐illumination rise in [K^+^]_o_, for each opsin individually. The *R* ^2^ value, which is a measure of the amount of the variance in the dependent variable (the [K^+^]_o_ rise) explained by the variance in the independent variable (the [K^+^]_o_ drop), was low, and the *p* values indicate that the level of association was no different from chance. Since the independent variables for the two opsins were almost non‐overlapping for these baseline datasets (Figure 3a), we extended our analyses by including also recordings made in the presence of TEA, which lowered both the illumination‐induced [K^+^]_o_ drop and the post‐illumination [K^+^]_o_ rise (Figure 3b). With this extended dataset, we saw an increase in the correlations for the two opsins separately. Pooling both opsins together as a single dataset (Figure 3c) increased the *R* ^2^ value. Notably, the best fit for the pooled data set yielded residuals in which every archaerhodopsin slice, except one, was negative (Figure 3c inset). There was a highly significant difference in the residuals, for this optimal fit, between the opsins, indicating that the two opsins should not be considered to behave as a single population in this analysis. When the two opsins are treated as separate categories (Figure 3d), there was a further marked increase in *R* ^2^, indicative of a substantially improved model. The model can be further improved by allowing halorhodopsin and archaerhodopsin to have different slopes (equivalent to Figure 3b; *R* ^2^ = 0.706). This optimal model thus suggests that the post‐illumination surge reflects both the initial influx and the choice of opsin. That is to say, the more K^+^ that goes in, the more has to come out, but this post‐illumination surge is additionally boosted for slices expressing halorhodopsin. Thus, the variance of the post‐illumination rise cannot be explained simply by the variance in the preceding drop in [K]_o_ but must incorporate an additional effect of which opsin is used. Interestingly, when the KCC2 blocker, VU 0463271, was applied to halorhodopsin‐expressing slices, it appeared to decouple the association between the [K^+^]_o_ drop and the [K^+^]_o_ rise (Figure 4), with a highly significant difference between the optimal single and optimized paired regressions (*F*_[1,27]_ = 0.022; *aoctool.m*).

A final point of difference is that in none of the archaerhodopsin experiments did we observe CSD events; in contrast, these were triggered quite readily in halorhodopsin‐expressing tissue (not illustrated, but examples are found in Parrish et al., 2023).

## 4 DISCUSSION

Archaerhodopsin and halorhodopsin have both been used to inhibit neuronal activity, but do so by driving ionic movement in opposite directions: halorhodopsin by driving Cl^−^ inwards, and archaerhodopsin by driving H^+^ out. We show here that this is further reflected by differences in the pattern of secondary effects, consistent with our interpretation of these effects reported in a previous study of halorhodopsin (Parrish et al., 2023). The two optogenetic pumps share the effect of hyperpolarizing neurons, and consistent with this, both show a secondary drop in [K^+^]_o_, caused by K^+^ moving into the stimulated cells through leak channels driven by the altered electrochemical balance for K^+^ during the period of opsin activity. Although we have used a somewhat unusual opsin activation protocol to show this effect (prolonged illumination, instead of the more typically used short pulses), it is important to realize that the ionic redistribution is through open channels and thus very fast, so would be expected to be seen also with short pulses, particularly sustained trains of these. The intervals between the pulses, though, will allow some recovery toward the baseline, and may also show overshoot of [K^+^]_o_.

The rebound, post‐illumination surge in [K^+^]_o_, however, appeared much smaller for archaerhodopsin, even allowing for the fact that the expression levels of the two opsins were not exactly equivalent (halorhodopsin expression was achieved by cross‐breeding Emx1 and floxed halorhodopsin mouse lines; archaerhodopsin expression was obtained by injecting wild‐type mice with a viral vector carrying the archaerhodopsin gene) and so we were unable to achieve the same degree of K^+^ loading during the periods of illumination.

The much larger rebound surge in [K^+^]_o_ with halorhodopsin is, in fact, in line with what can be predicted from the different actions of the two opsin pumps. Halorhodopsin loads the cell with chloride (Zhang et al., 2007), whereas archaerhodopsin pumps protons out of the cell (Chow et al., 2010). Even if halorhodopsin and archaerhodopsin current amplitudes and durations were identical, only halorhodopsin loads the cell with parallel inward fluxes of K^+^ and Cl^−^, leading to outward transport of KCl by KCC2, which is an electroneutral transporter capable of restoring a normal intraneuronal Cl^−^ level rapidly after a Cl^−^ load. Notably, blocking KCC2 in halorhodopsin‐expressing brain slices appeared to decouple the relationship between the drop and the post‐illumination rise (Figure 4). This pattern of ionic redistribution is consistent with previous observations that the rebound [K^+^]_o_ surge, following chloride‐loading arising either naturally, or following intense GABAergic activation, is reduced by blockers of KCC2 (Viitanen et al., 2010).

These collected observations further reinforce the view that [Cl^−^]_i_ and [K^+^]_o_ are tightly coupled, as seen with the large rise in [K^+^]_o_ that follows intense interneuronal activation or direct GABA application (Chang et al., 2018; Viitanen et al., 2010). These observations warrant a reconsideration of the nature of raised [K^+^]_o_ during epileptic seizures, which may comfortably exceed 10 mM at its peak (Hablitz & Heinemann, 1987; Librizzi et al., 2017; Raimondo et al., 2015; Somjen, 2004). This has often been attributed solely to K^+^ movement out of cells during action potentials, as described originally by Hodgkin and Huxley (Hodgkin & Huxley, 1952). Recordings of the ictal initiation period typically also show a significant rise in [K^+^]_o_ ahead of the time when pyramidal cells are recruited, as is readily apparent in published recordings (Chizhov et al., 2022; Librizzi et al., 2017). Notably, this early peak coincides with a period of very intense interneuronal activity (Parrish et al., 2019; Trevelyan et al., 2006). A major contribution to this early rise, therefore, is likely to be the extrusion of K^+^ coupled to Cl^−^‐clearance, following a period of significant chloride‐loading due to intense GABAergic bombardment. Recognizing these two different causes of K^+^ extrusion—(1) action potential associated with K^+^ conductance and (2) transporter‐mediated efflux, coupled to the clearance of Cl^−^—is important to our understanding of ictal propagation and recruitment to seizures (Graham et al., 2022; Trevelyan et al., 2022).

The most surprising secondary effect triggered by prolonged halorhodopsin activation was the occurrence of CSDs, at times, arising from a state where the majority of neurons were hyperpolarized to different degrees, and [K^+^]_o_ was below baseline levels (Parrish et al., 2023). We did not observe such events in our archaerhodopsin preparations. Two possible explanations suggest themselves for this difference, both consistent with our data: first that the lack of CSDs in our archaerhodopsin experiments reflects the smaller shifts in K^+^ (Figure 3), or second that it reflects differences in the net ion flux during illumination. Halorhodopsin activation results in both inward Cl^−^ and K^+^ movement, generating a large net influx of osmotically active particles. In contrast, archaerhodopsin drives an outward proton movement, and the K^+^ redistribution is much smaller. While this negative finding (no CSDs in our archaerhodopsin experiments) represents a limited test of the hypothesized differences between halorhodopsin and archaerhodopsin, the result is consistent with what else is known about their different mechanisms of action.

## AUTHOR CONTRIBUTIONS

The project was conceived by R. Ryley Parrish, Juha Voipio, and Andrew J. Trevelyan. The rig was installed by R. Ryley Parrish and Juha Voipio. Experiments were performed by R. Ryley Parrish and Tom Jackson‐Taylor. Analyses were performed by R. Ryley Parrish, Tom Jackson‐Taylor, and Andrew J. Trevelyan. The manuscript was written by Andrew J. Trevelyan, and all authors provided edits.

## FUNDING INFORMATION

The work was supported by grants from BBSRC (BB/P019854/1) and MRC (MR/R005427/1).

## CONFLICT OF INTEREST STATEMENT

The authors declare no conflicts of interest.

## ETHICS STATEMENT

All procedures performed were in accordance with the guidelines of the Home Office UK and Animals (Scientific Procedures) Act 1986 and approved by the Animal Welfare and Ethical Review Body at both Newcastle University. Male and female mice were used for experimentation.
